# Supplementary material for: Using X-ray tomoscopy to explore the dynamics of foaming metal
Source: Nat Commun. 2019 Aug 21;10:3762. doi: 10.1038/s41467-019-11521-1 (PMC6704127; doi:10.1038/s41467-019-11521-1)
Supplement: Supplementary file 1 — Supplementary Information [file 41467_2019_11521_MOESM1_ESM.pdf]

Using X-ray tomoscopy to explore the dynamics of foaming metal

García-Moreno et al.

Supplementary Figures

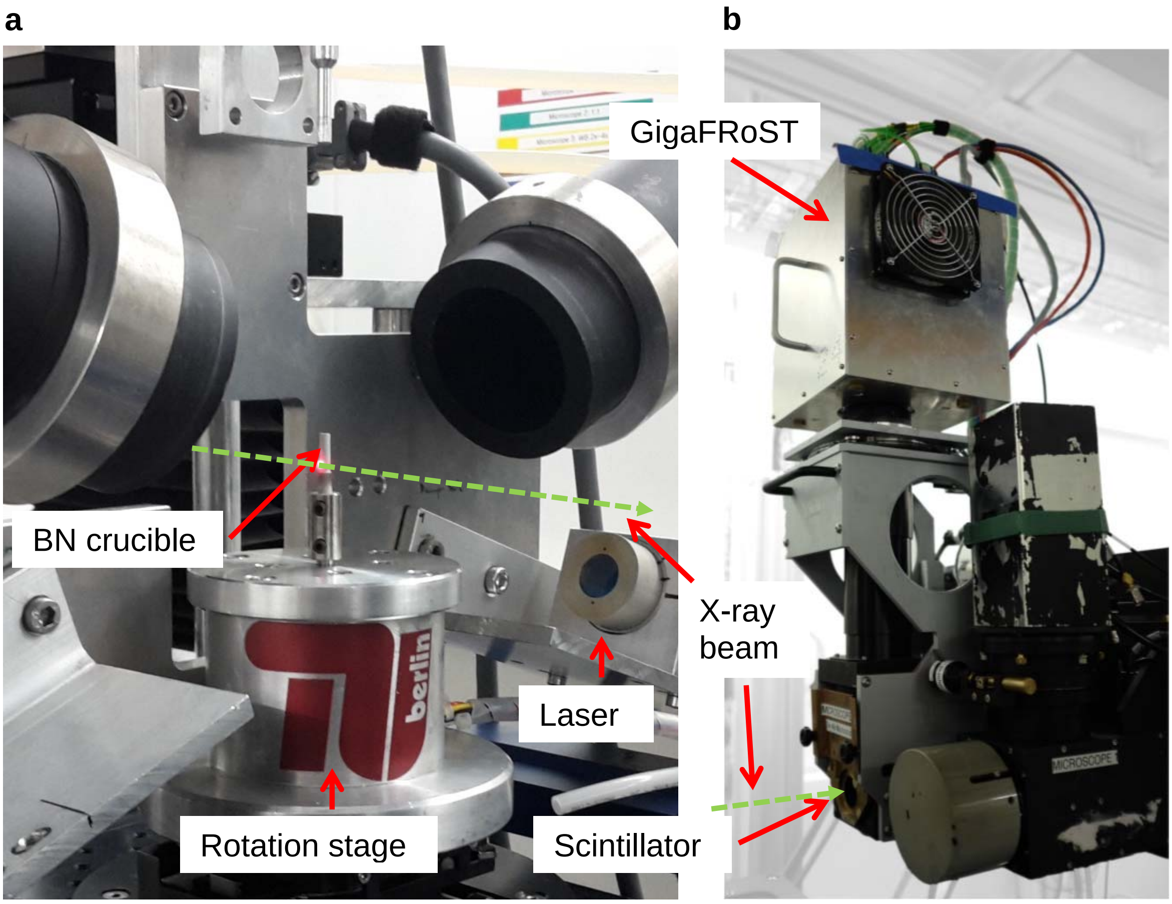

Supplementary Figure 1. **Image of the tomoscopy setup** used for investigating foaming aluminium alloys. **a**, Fast rotation stage (>100 s^{-1} (revolutions per second), allowing for acquisition rates >200 tps) and a cylindrical crucible made from boron nitride with a foamable sample inside. The foaming temperature was provided by two 150 W near-infrared lasers and the temperature reading by a calibrated pyrometer. **b**, Optical microscope with the scintillator screen on the bottom part and the GigaFRoST CMOS camera on top

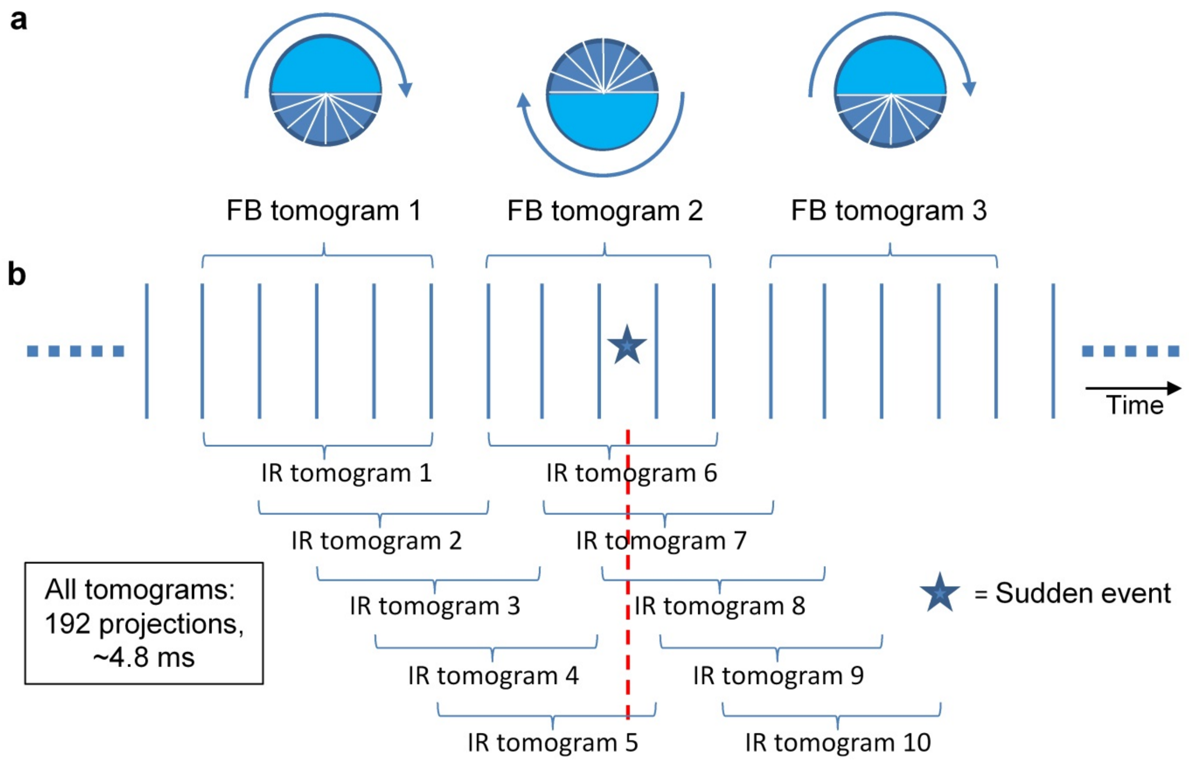

Supplementary Figure 2. **Principle of incremental reconstruction. a**, Fixed-block (FB) tomograms. **b**, Incrementally reconstructed (IR) tomograms with a floating start time. Each vertical dash denotes one radiographic projection. The star and the red broken line mark a sudden change of the sample

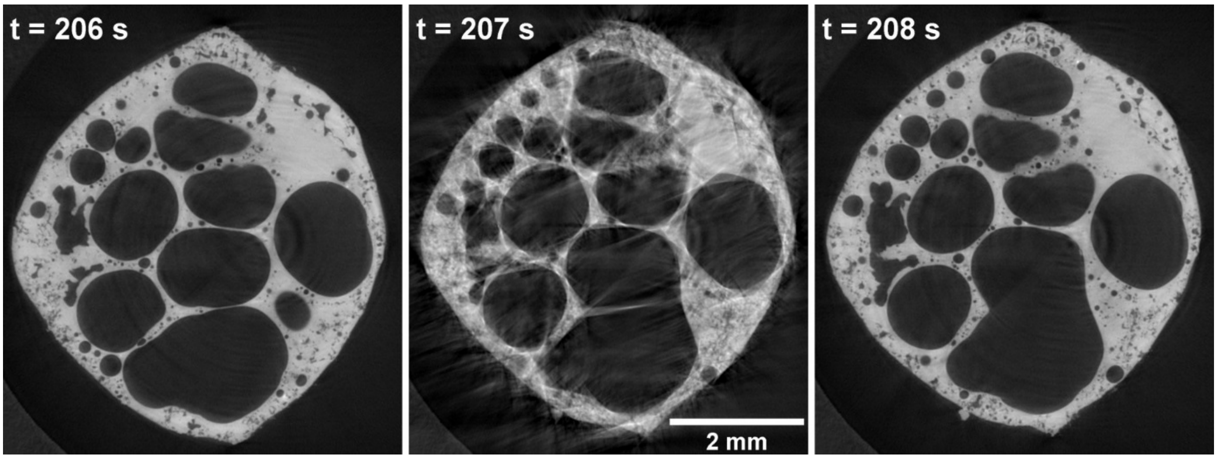

Supplementary Figure 3. **Motion artefacts** occurring in the image sequence acquired at 1 tps as reconstructed with fixed blocks (see Supplementary Figure 2) and shown in Figure 2

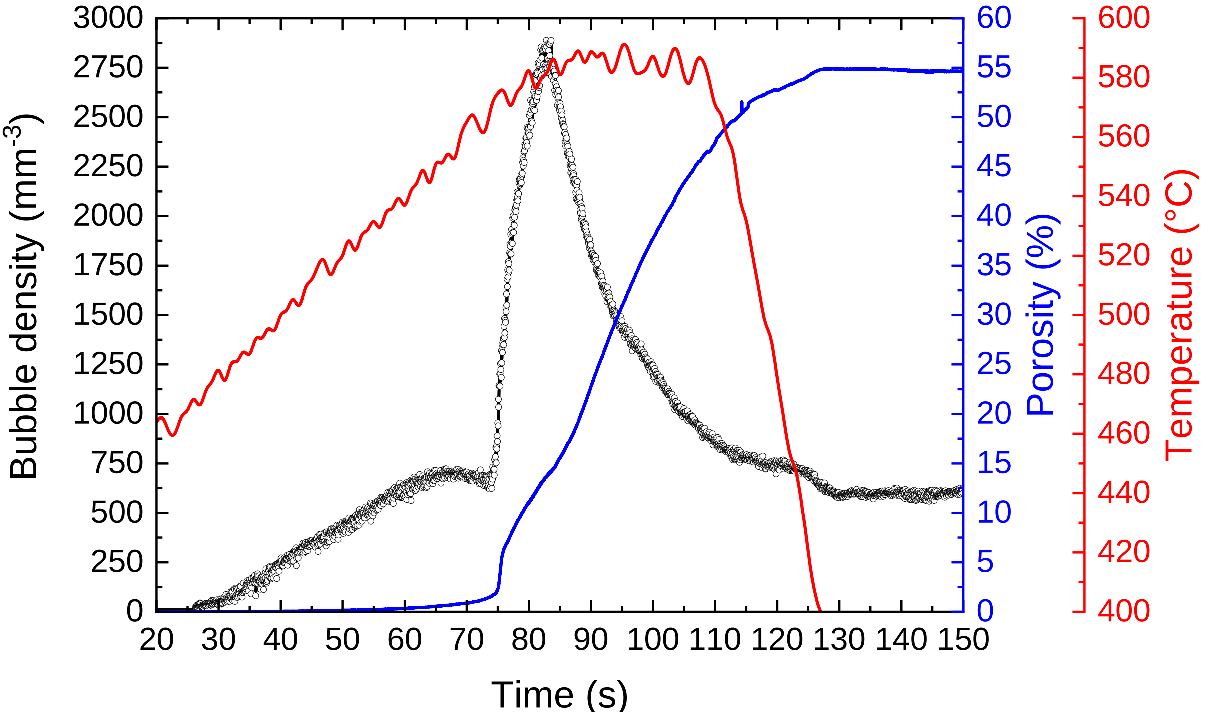

Supplementary Figure 4. **Foam evolution characterised by tomoscopy** similar to experiment in Figure 2 but carried out at an acquisition rate of 20 tps, thus avoiding motion artefacts. Note that the bubble number systematically decreases for longer foaming times due to expansion of the foam to beyond the field of view

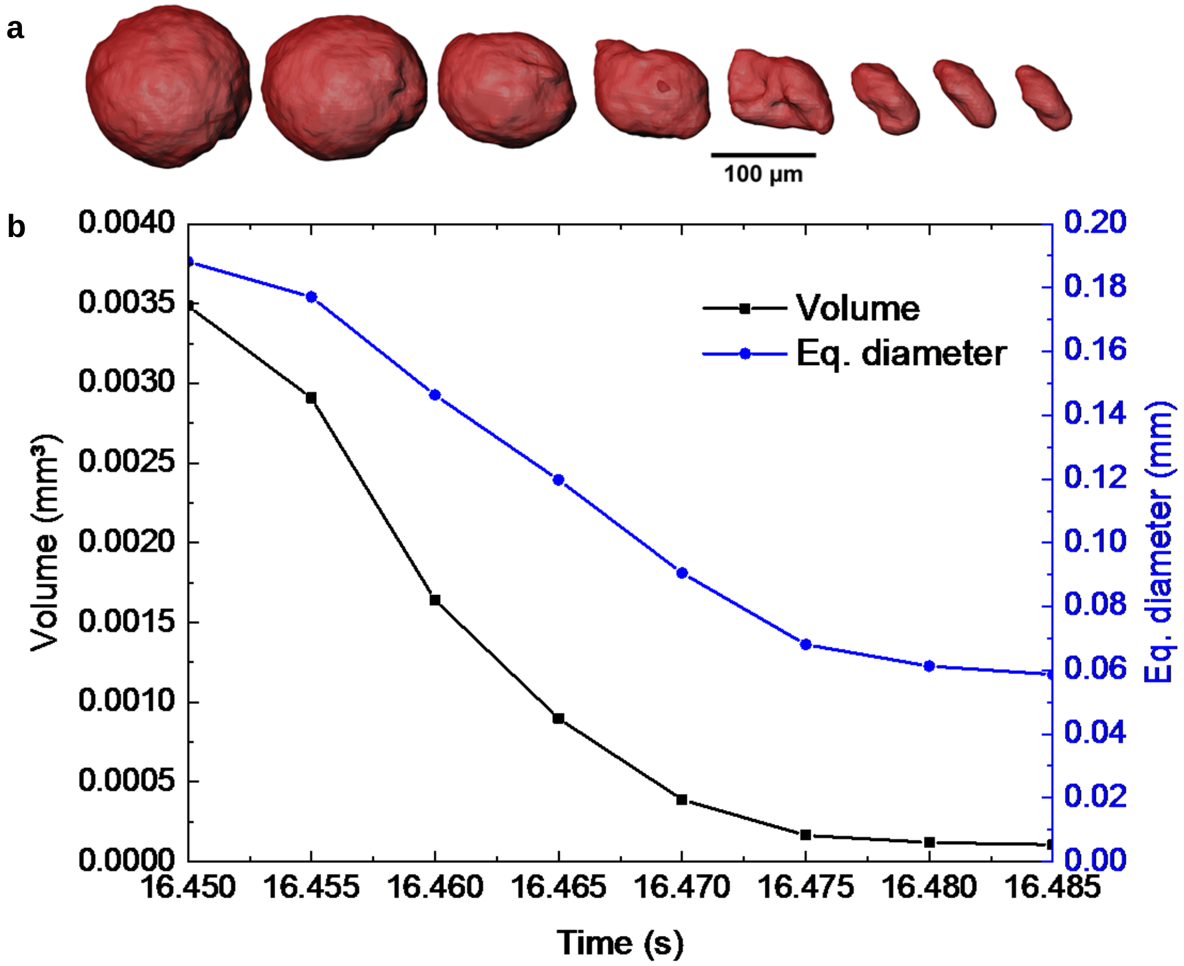

Supplementary Figure 5. **Collapse of the bubble** shown in red in Figure 4. **a**, Visualisation of bubble evolution. **b**, Quantification of bubble volume

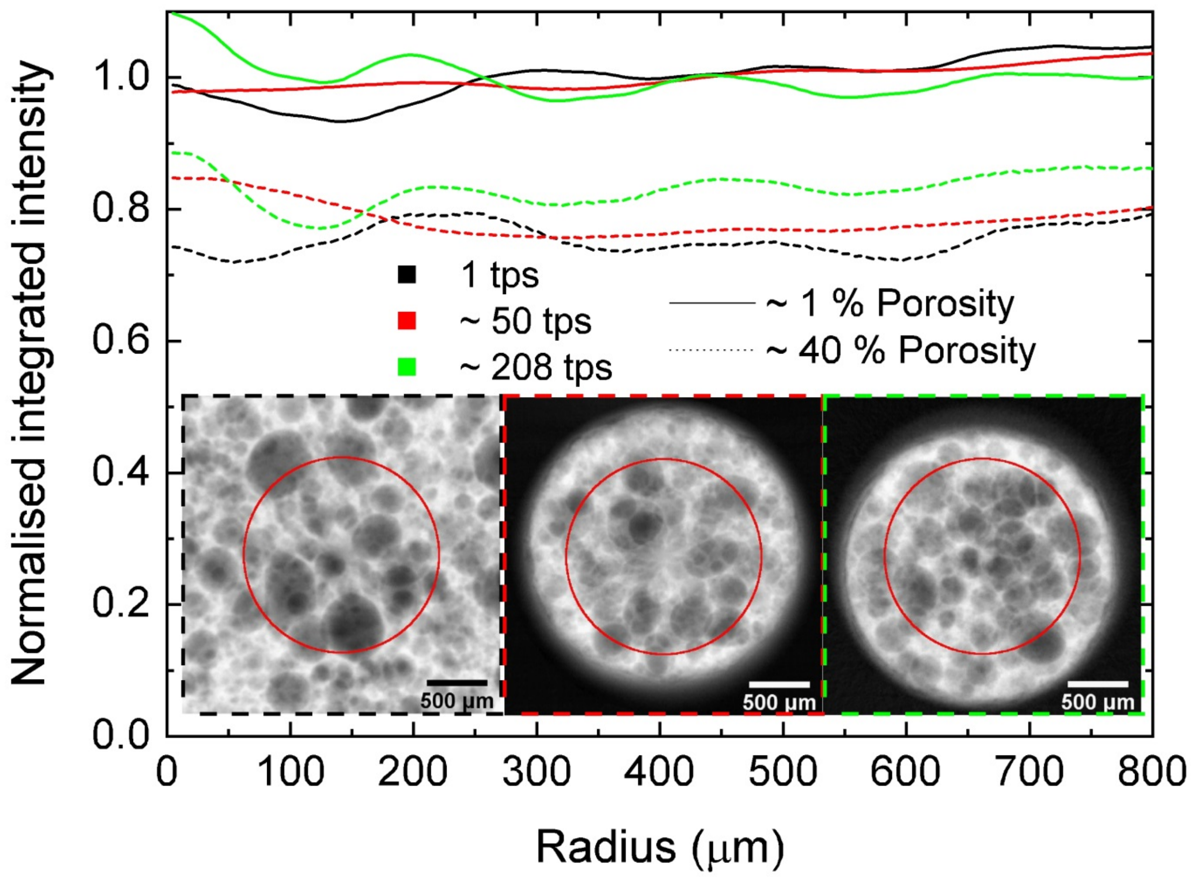

Supplementary Figure 6. **Radial density profiles** shown as normalised integrated intensities over the radial coordinate of different cylindrical liquid foams investigated by tomoscopy at 1 tps (max. 0.001 g radial acceleration), 50 tps (max. 2.5 g) and 208 tps (max. 43 g). Solid and dashed curves denote the results for early (1% porosity) and later foaming stages (40% porosity), respectively. Insets show reconstructed slices superimposed over the z axis of the foams at ~40 % porosity. Regions of interest are located inside the red circles for the three acquisition rates applied (as colour coded)

Supplementary Tables

Quantity	Value
Tomoscopy rate	208 tps
Time per tomogram	4.8 ms
Exposure time	0.02 ms
Exposure period	0.025 ms
Frame rate of radiograms	40 kHz
Frames per tomography	192
Number of frames in 1 min of acquisition	2.4×10^6
Number of tomograms in 1 min of acquisition	12,500
Effective pixel size	4.9 µm
Spatial resolution	≤15 µm
Image size (h × v)	528 × 128 pixels
Field of view (h × v)	2.61 × 0.63 mm^2

Supplementary Table 1. **Important parameters** characterising tomoscopy experiment carried out at the highest acquisition rate
