# Supplementary material for: Using X-ray tomoscopy to explore the dynamics of foaming metal
Source: Nat Commun. 2019 Aug 21;10:3762. doi: 10.1038/s41467-019-11521-1 (PMC6704127; doi:10.1038/s41467-019-11521-1)
Supplement: Supplementary file 9 — Description of Additional Supplementary Files [file 41467_2019_11521_MOESM9_ESM.docx]

Description of Additional Supplementary Files

**Supplementary Movie 1** corresponding to Figure 2a-d: Surface rendering of the evolving metal foam. Acquisition rate is 1 tps, video plays 5x faster than in reality

**Supplementary Movie 2** corresponding to Figure 2e: TiH2 particles (shown in blue) are seen to inflate bubbles locally after which films rupture. Acquisition rate is 1 tps, video plays 5x faster than in reality

**Supplementary Movie 3** corresponding to Figure 3: Tomographic slices of film rupture between two bubbles. Acquisition rate is 208 tps, video plays 20x slower than in reality

**Supplementary Movie 4** corresponding to Figure 4: 3D rendering of bubble evolution (full field of view) of the foam. Acquisition rate is 208 tps, video plays 10x slower than in reality Francisco Garcia-Moreno Institut für Angewandte Materialforschung Unser Zeichen: Hahn-Meitner-Platz 1 14109 Berlin Tel +49 30 8062-42761 Fax +49 30 8062-43059 garcia-moreno@helmholtz-berlin.de Ihr Zeichen: Bearbeiter/in: Berlin, 05.07.2019 Seite 2

**Supplementary Movie 5** corresponding to data in Figures 3, 4 and 6: 3D rendering of overall bubble evolution (full field of view) of the foam. Acquisition rate is 208 tps, but only every 100th tomogram was used for the video, video plays 2x slower than in reality

**Supplementary Movie 6** corresponding to Figure 5: Radioscopic quantification of foam stability by counting accumulated rupture events during isothermal holding of liquid metallic foams with (left) and without (right) blowing agent. Radiography acquisition rate is 1 fps, video plays 5x faster than in reality

**Supplementary Movie 7** corresponding to Figure 6: 3D visualisation of incrementally reconstructed collapsing bubbles. Features touching the edges were removed, which induces the deletion of some features in individual frames and additional artefacts. Tomography repetition rate is 40,000 s-1 , video plays 1000x slower than in reality
